# Serous Hæmorrhage

**Published:** 1908-05-30

**Authors:** 


### SEROUS HÆMORRHAGE.

Sir Almroth Wright and his school believe that in states of lowered coagulability of the blood, dependent on relative deficiency of its calcium salts, there is a tendency for serous exudations to occur. They think that these exudations may affect various tissues, and so give rise to various symptoms, and that in this way several conditions not at present associated may be found to have a common explanation.

On this theory, for instance, nettle-rash consists in a serous exudation, or "serous hæmorrhage," into the skin, with production of the characteristic urticarious wheals: adolescent physiological albuminuria, in exudation into the renal tubules, with production of albumen in the urine. It is stated that in both affections there is to be found a low lime-content, and diminished coagulability, of the blood, the result of a decalcifying process; in the first instance a chemical one—by excess of acids, ingested through errors in diet—and in the second mechanical, through the heavy call on the system for lime-salts which a period of rapid bone-formation must inevitably entail. In either, then, the chief indication for treatment is considered to be the replenishing of the blood with lime, as by exhibition of the lactate, or chloride, of calcium. And certainly in both cow's milk, which is rich in lime-salts, has long been known to be a good diet.

All this, if true, is clearly a novel, valuable, and unusually definite piece of pathology. There may be some interest, therefore, in examining the wealth of deduction with which Wright surrounds his theory and endeavours to strengthen the chain of reasoning briefly indicated above. He holds that acid drinks (a surfeit of which is known to be capable of bringing on urticaria in predisposed subjects), assuage thirst by decalcifying the blood and thereby provoking a flushing of the tissues with serum. He recalls that an old-fashioned poultice for "drawing" boils was of soap and sugar—soap, a powerful decalcifier, as all who have used hard water for washing purposes are aware, and sugar, which promotes osmosis. The empirical virtue of this application is explained by the action of the soap in inducing, and of the sugar in maintaining, an increased flow of lymph in the diseased area, thus bringing up new supplies of bacteriotropic substances to be expended in the strife with micro-organisms.

Generally, however, there is more opportunity for calcium therapy in the checking of local exudations, as in the instances first given; and it is claimed that a weeping surface has been observed to dry up, and the pain of chilblains to be relieved, after lime-salts have been given by the mouth. One of Wright's fellow-workers believes that he has made a complete demonstration of the whole process in the case of a certain form of cephalalgia associated with low blood coagulability, and conjectured to be dependent on intra-cranial exudation. The symptoms abated after a dose or two of calcium lactate, and coincidently the coagulability and the calcium content of the blood rose. They fell again, however, and the headache reappeared, when sodium citrate was given. Now sodium citrate will be remembered as in use in laboratories to keep blood from clotting; that it does so by its decalcifying action is almost certain.

The unifying character of the whole conception is obvious, although the ingenious experiments in proof require independent confirmation before their results can be fully accepted. These and other studies on blood coagulation, though only part, as it were, of the *parerga* of this distinguished investigator, will perhaps be found to have a more immediate practical application than the system of vaccine therapy peculiarly associated with his name.

